# Novel antibodies against GPIbα inhibit pulmonary metastasis by affecting vWF-GPIbα interaction

**DOI:** 10.1186/s13045-018-0659-4

**Published:** 2018-09-17

**Authors:** Yingxue Qi, Wenchun Chen, Xinyu Liang, Ke Xu, Xiangyu Gu, Fengying Wu, Xuemei Fan, Shengxiang Ren, Junling Liu, Jun Zhang, Renhao Li, Jianwen Liu, Xin Liang

**Affiliations:** 1 State Key Laboratory of Bioreactor Engineering & Shanghai Key Laboratory of New Drug Design, School of Pharmacy, East China University of Science and Technology, 130 Meilong Rd, Shanghai, 200237 People’s Republic of China; 2 Aflac Cancer and Blood Disorders Center, Children’s Healthcare of Atlanta, Department of Pediatrics, Emory University School of Medicine, Atlanta, GA 30322 USA; 3 Central laboratory, General Surgery, Putuo Hospital, and Interventional Cancer Institute of Chinese Integrative Medicine, Shanghai University of Traditional Chinese Medicine, 164 Lanxi Rd, Shanghai, 200062 People’s Republic of China; 4 Department of Medical Oncology, Shanghai Pulmonary Hospital, Thoracic Cancer Institute, Tongji University School of Medicine, Shanghai, People’s Republic of China; 5 Department of Biochemistry and Molecular Cell Biology, Shanghai Key Laboratory of Tumor Microenvironment and Inflammation, Shanghai Jiao Tong University School of Medicine, Shanghai, 200025 People’s Republic of China; 6 Division of Hematology, Oncology and Blood & Marrow Transplantation, Department of Internal Medicine, Holden Comprehensive Cancer Center, University of Iowa Carver College of Medicine, Iowa City, IA 52242 USA

**Keywords:** GPIbα, vWF, Platelets, Antibody, Metastasis

## Abstract

**Background:**

Platelet glycoprotein Ibα (GPIbα) extracellular domain, which is part of the receptor complex GPIb-IX-V, plays an important role in tumor metastasis. However, the mechanism through which GPIbα participates in the metastatic process remains unclear. In addition, potential bleeding complication remains an obstacle for the clinical use of anti-platelet agents in cancer therapy.

**Methods:**

We established a series of screening models and obtained rat anti-mouse GPIbα monoclonal antibodies (mAb) 1D12 and 2B4 that demonstrated potential value in suppressing cancer metastasis. To validate our findings, we further obtained mouse anti-human GPIbα monoclonal antibody YQ3 through the same approach.

**Results:**

1D12 and 2B4 affected the von Willebrand factor (vWF)-GPIbα interaction via binding to GPIbα aa 41-50 and aa 277-290 respectively, which markedly inhibited the interaction among platelets, tumor cells, and endothelial cells in vitro, and reduced the mean number of surface nodules in the experimental and spontaneous metastasis models in vivo. As expected, YQ3 inhibited lung cancer adhesion and demonstrated similar value in metastasis. More importantly, for all three mAbs in our study, none of their Fabs induced thrombocytopenia.

**Conclusion:**

Our results therefore supported the hypothesis that GPIbα contributes to tumor metastasis and suggested potential value of using anti-GPIbα mAb to suppress cancer metastasis.

**Electronic supplementary material:**

The online version of this article (10.1186/s13045-018-0659-4) contains supplementary material, which is available to authorized users.

## Background

The association between elevated platelet number and malignant tumors was initially reported in 1872 [1, 2] and has been demonstrated in several common cancers [3–6]. Tumor cells are capable of activating and aggregating platelets to form tumor thrombus—a process referred to as tumor cell-induced platelet aggregation (TCIPA) [7]. Extensive evidence indicates that the formation of tumor thrombus contributes to critical steps in cancer metastasis, including shielding cancer cells from physiological clearance and immune surveillance and facilitating the migration, invasion, and arrestment of tumor cells within the vasculature [2, 8]. It is increasingly recognized that the formation of tumor thrombus involving platelets is the first and one of the most important steps in cancer metastasis.

Two important platelet membrane receptors, glycoprotein Ib-IX-V (GPIb-IX-V) and glycoprotein IIb-IIIa (GPIIb-IIIa, also known as integrin α_IIb_β_3_), are essential for tumor cell-platelet adhesion and aggregation when tumor cells invade into vasculature [2]. An increasing number of studies have focused on the role of platelet membrane receptors in tumor metastasis [7, 9–11]. Although it is generally believed that the deficiency of GPIIb-IIIa or blockade of GPIIb-IIIa by monoclonal antibodies may lead to severe bleeding complications [11], which is the main reason to limit the clinical use of these anti-GPIIb-IIIa agents in cancer therapy, the anti-metastatic agent anti-GPIIIa49-66 scFv Ab A11 that has a slight effect on platelet count and vein bleeding time was found to have therapeutic potential in metastasis [7, 12]. While the mechanism of GPIIb-IIIa involvement in tumor metastasis is largely clarified, the role of another important adhesion receptor GPIb-IX-V in metastasis remains debatable [13]. Here, we evaluated the role of GPIb-IX-V and its therapeutic potential in metastasis.

The GPIb-IX-V complex consists of four subunits: GPIbα, GPIbβ, GPIX, and GPV. It interacts with many important extracellular ligands. GPIbα is the largest and most important component of the complex. The N-terminal domain of GPIbα contains the binding sites for several molecules, including vWF [14], P-selectin (CD62P) [15], and thrombin [16], which are essential for primary hemostasis and blood coagulation. The interaction between vWF and GPIbα was found to be particularly critical in the formation of thrombus [17]. Although there are studies that showed that knocking out the mouse GPIbα or replacing mouse GPIbα extracellular domain could significantly inhibit tumor cell metastasis [18], the deletion of GPIbα extracellular domain unfortunately induced platelet depletion, leading to severe bleeding complications [18]. In addition, blockage of GPIbα by monoclonal antibody p0p/B did not have the same influence on tumor metastasis as GPIbα knock out models [9], raising the concern if GPIbα truly participates in the metastatic process. To address this question, we screened out three anti-GPIbα mAbs with minimal effect on platelet activation as the tools to dissect the therapeutic value of GPIbα in cancer metastasis.

## Methods

### Materials and animals

Platelet agonist ADP and collagen (equine tendon) were from HELENA laboratories (USA). Ristocetin was from Sigma (R7752, USA). Anti-human GPIbα monoclonal antibody SZ2, VM16d, and AK2 were from GenTex (GTX28822, USA), YO Proteins (656, USA), and Bio-Rad (MCA740T, USA), respectively. Secondary antibody anti-human/mouse CD62P (P-selectin) APC was from Thermo Fisher scientific (17-0626, USA), and FITC-conjugated anti-human PAC-1 was from Biolegend (362803, USA). Peptides of GPIbα fragments were synthesized by GL Biochen (China) Ltd. Recombinant mouse vWF protein was from Creative BioMart (VWF-1432 M, USA), and human vWF protein was from Sino Biological (10973-H08C, USA). C57BL/6J mice, BCLB/C mice, and Wistar rat were from JSJ laboratories (China) and were bred and housed at Putuo animal care facility. Transgenic mice expressing no mouse but only human GPIbα (hTg) were described previously [19]. Animal experiments were conducted in mice or rats using protocols approved by the IACUC of Putuo Hospital. Six- to eight-week-old mice or rats were used in the study, and investigators were blinded to group allocation during data collection.

### Human blood collection

Written, informed consent was obtained from all participants prior to their inclusion in studies. Venous blood was collected from healthy adult volunteers at East China University of Science and Technology, as well as lung cancer patients at Shanghai Pulmonary Hospital. In addition, the use of donor-derived human platelets was approved by IRB in Shanghai Pulmonary Hospital.

### Production and characterization of GPIbα mAbs

Rat anti-mouse GPIbα monoclonal antibodies and mouse anti-human GPIbα monoclonal antibodies were prepared according to the methods described by Koehler and Milstein [20]. Briefly, to develop mouse anti-human antibodies, BALB/C female mouse (6 to 8 weeks) were immunized by four injections of human platelet lysate at a 28-day interval. To develop rat anti-mouse antibodies, Wistar female rats were given an intraperitoneal injection of washed mouse platelets four times at a 20-day interval. Three days after the fourth immunization, mouse or rat splenocytes were fused with Sp2/0-Ag14 myeloma cells and cultured in HAT selection medium. IgGs were purified from hybridoma supernatants using a protein G-Sepharose 4B column (6518-1, Biovision, CA, USA).

The ELISA assay was then used to characterize these antibodies. Ninety-six well microtiter plates were coated overnight at 4 °C with 50 μl of 4 μg/ml anti-human/mouse CD42a Ab (MBS9206081, MyBioSource). After washing three times, the wells were blocked with 2% (*w*/*v*) BSA-PBS for 2 h. The wells were then incubated with lysate of human or mouse platelets for 2 h. After that, hybridoma supernatants or purified mAbs were added to the wells and incubated for 60 min. The bound antibodies were detected by HRP-conjugated goat anti-mouse IgG or goat anti-rat IgG. After the background signal had been subtracted, the binding curve was fitted to the eq. *Y* = *B*_max_ × [ligand]/(*K*_d_ + [ligand])

where *Y* is the specific binding, [ligand] the ligand concentration, *B*_max_ the binding maximum, and *K*_d_ the equilibrium dissociation constant.

The specificities of the antibodies were also determined by Western blot as previously described [21].

### Preparation of Fab fragment

The generation of Fab fragment was following previously described protocol with the modification in incubation time with immobilized papain (20341, Thermo Scientific) [21]. After papain was removed via centrifugation, the generated Fab fragment was purified using Protein A beads (6501-5, Biovision, CA, USA).

### Platelet activation

Washed platelets (1.2 × 10^7^ cells/ml) were treated with hybridoma supernatants or purified mAbs at room temperature (RT) for 20 min and detected with FITC or APC-conjugated antibody. Platelet activation induced by tumor cells was by adding 1 × 10^5^ cells/100 μl tumor cells to 1.2 × 10^6^ cells/100 μl washed platelets. When needed, the antibody was added to the platelets and incubated for at least 20 min before their stimulation by tumor cells. The signal of platelet activation was quantitated by the mean fluorescence intensity for the entire cell population (10,000 cells) on a Becton-Dickinson FACS Canto II instrument (BD Biosciences, San Jose, CA, USA).

### Platelet aggregometry

Platelet-rich plasma (PRP) was generated as previously described [21]. The final platelet count in PRP was adjusted to 2.5 × 10^8^ cells/ml. Aggregation was initiated in 300 μl of stirred PRP by the addition of noted agonists or 1 × 10^5^ cells/50 μl MCF-7 cells to form the mixture of platelets and tumor cells. When required, the antibody was added to PRP and incubated for at least 5 min before stimulation with either agonists or tumor cells. Agonist-induced platelet aggregation was monitored in dual-channel Chrono-Log aggregometer (Havertown, PA, USA)

### Assay of tumor cells adhesion to platelets, platelet adhesion to endothelial cells, or tumor cell adhesion to endothelial cells

The adhesion between tumor cells, platelets, and endothelial cells was measured as previously described [7].

### Animal experiments

#### Experimental lung metastasis assay

In the Lewis lung carcinoma (LLC) model, 6-week-old C57BL/6J mice were randomly divided into six groups. There were eight mice per group, and half of the mice are male and half are female in each group. Female and male mice were separately cultured to avoid mating. For groups 1–6, mice were injected with LLC tumor cells (2.5 × 10^5^ cells/mouse) with control IgG, control Fab, 2B4, 1D12, 2B4 Fab, or 1D12 Fab, respectively, at the dose of 50 μg/mouse through the lateral tail vain, along with the tumor cells. After 14 days, the lungs were removed, rinsed with PBS, and the number of metastatic foci on the lung surface was counted. The pulmonary lobes were subsequently kept in 4% paraformaldehyde for later paraffin embedding and hematoxylin and eosin staining. The same experiment was repeated using B16F10 melanoma mouse model (1 × 10^6^ B16F10 cells/mouse).

#### Spontaneous metastasis assay

For spontaneous metastasis, 6-week-old BCLB/C female mice were subcutaneously injected with 1 × 10^5^ 4T1 tumor cells. The mice were treated with 50 μg/mouse 2B4, 2B4 Fab, 1D12, or 1D12 Fab, respectively, when the tumor volume reached 80 mm^3^. After 3 weeks, the mice were killed, and the surface metastatic nodules on the lung were counted and the volume of primary tumor was recorded.

#### Determination of mouse platelet count

The platelet number was quantitated by a Becton-Dickinson FACS Canto II instrument equipped with BD Trucount Tubes (340334). The subsequent steps were then carried out by following the manufacturers’ instructions.

#### Bleeding time

The bleeding time was measured as previously described [22]. Briefly, the mouse tail vein was severed 2 mm from its tip and blotted every 30 s on a circular sheet of filter paper to obtain an objective measurement. Bleeding time was calculated when there was absence of blood on the filter paper. Bleeding time differences were recorded by an unbiased observer and confirmed by two other observers blinded to the experimental status of the mice.

### Statistical analysis

Statistical analysis was performed using Prism 6 software. All experiments were carried out at least three times, and the results are presented as the mean ± standard deviation. Statistical significance was assessed by using the one-way analysis of variance (ANOVA) followed by Dunnett’s post hoc test. *P* values < 0.05 were considered statistically significant.

## Results

### Generation and screening of mAbs targeting mouse platelet GPIbα

**Fig. 1:**
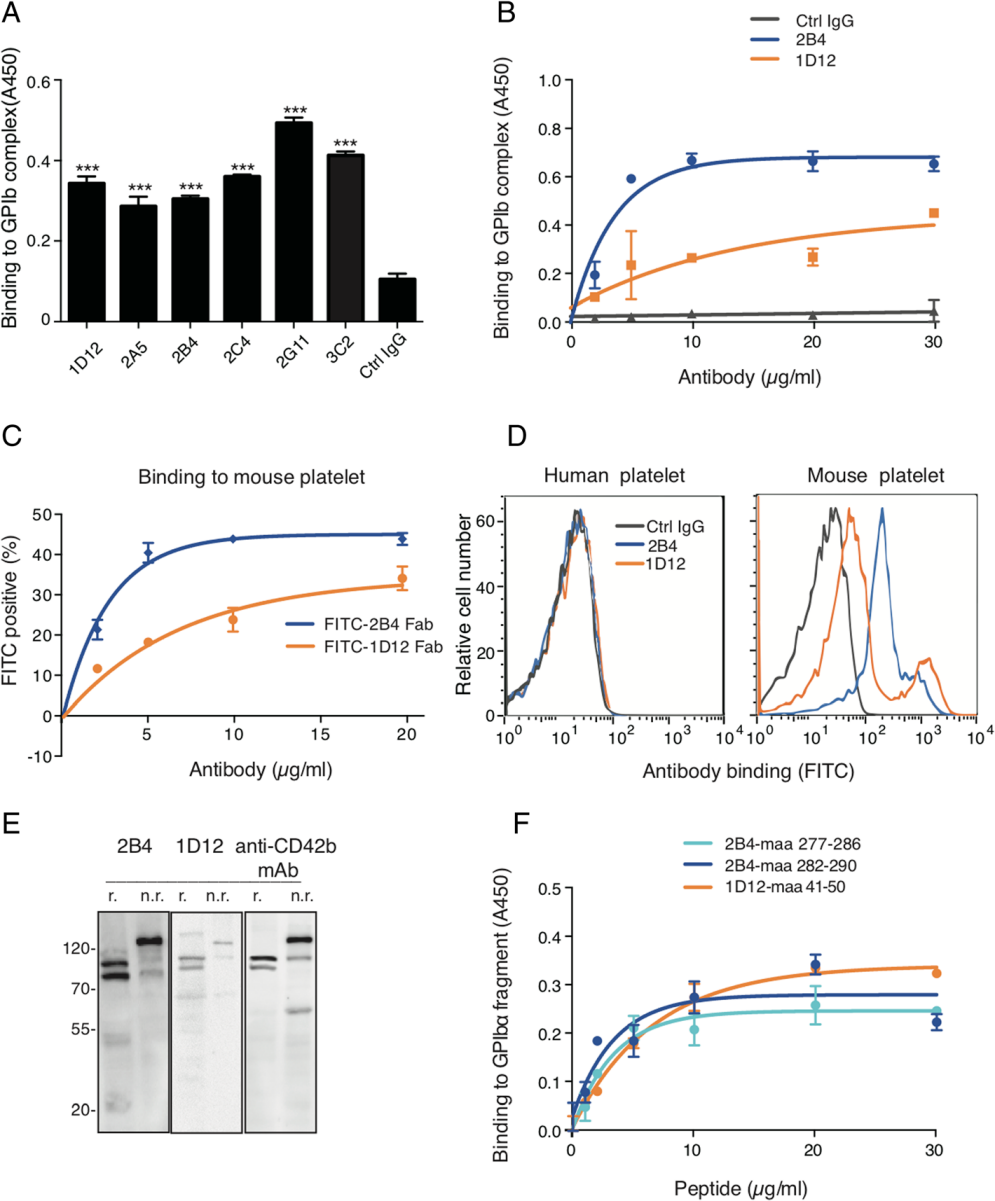
2B4 and 1D12 specifically bind to mouse glycoprotein (GP)Ibα. **a** Binding of rat anti-mouse antibodies to GPIb-IX complex was detected in ELISA. GPIb-IX was captured by anti-GPIX antibody which complex was immobilized in microtiter plates. Supernatant of hybridoma cells, each identified by the clone name, and the negative control, in the form of RPMI-1640 fetal bovine culture medium with 5 μg/ml rat IgG, were added to the coated wells. The bound Ab was detected with HRP-conjugated rabbit anti-rat IgG. ***P < 0.001. **b** Binding of 2B4 and 1D12 to GPIb-IX complex were detected in ELISA. Purified mAbs, colored as indicated, and negative controls, in the form of rat IgG, were added to the GPIb-IX immobilized microtiter plates. The bound Ab was detected with HRP-conjugated rabbit anti-rat IgG. **c** Binding of FITC-conjugated Fab to washed mouse platelets was detected in flow cytometry. Washed mouse platelets were incubated with each Fab at indicated concentration. Binding of Fab was detected by flow cytometry and quantitated by mean fluorescence intensity. **d** 2B4 and 1D12 specifically bound to mouse platelets. Washed human or mouse platelets were incubated with 10 μg/ml FITC-conjugated 2B4 or 1D12, respectively. Binding of Ab was detected by flow cytometry and quantitated by mean fluorescence intensity. **e** 2B4 and 1D12 recognized specifically GPIbα in Western blot under nonreducing (n.r.) and reducing (r.) conditions. Total lysates of mouse platelets were immunoblotted with either 2B4 or 1D12. Molecular weight marker (M) was shown and labeled in kDa on the left. **f** Binding of 5 μg/ml of 2B4 or 1D12 to indicated concentration of GPIbα peptide fragments were detected in ELISA. GPIbα peptide fragment was immobilized in microtiter plates. Indicated concentration antibodies were added to the coated wells. The bound Ab was detected with HRP-conjugated rabbit anti-rat IgG. Each figure or histogram is a representative of three independent experiments

To generate antibodies that bind to mouse platelet GPIbα, washed mouse platelet lysate was used as the antigen for rat immunization. Obtained hybridoma clones were screened in ELISA for binding affinity to the GPIb-IX complex. Positive clones were further screened for their abilities to inhibit platelet-cancer cell adhesion (Additional file 1: Table S1A). After screening, we obtained six positive clones that could bind to GPIb-IX complex (Fig. 1a) and inhibit platelet-tumor cell adherence to different extents (Additional file 2: Figure S1A). At static condition, two of the six antibodies, 2B4 and 1D12, had virtually no effect on the activation of integrin αII_b_β_3_, which is used to indicate platelet activation [21], while the other four could activate platelet to a certain degree in the same condition (Additional file 2: Figure S1B). Therefore, 2B4 and 1D12 were eventually selected for study.

Purified 2B4 and 1D12 (Additional file 2: Figure S1C) exhibited higher binding affinity to immobilized GPIb-IX complex than the negative control (Ctrl IgG) (Fig. 1b). The K_d_ of 2B4 and 1D12 was 2.47 ± 0.28 and 9.62 ± 0.52 μg/ml, respectively. Like 2B4 and 1D12 antibodies, their Fab fragments exhibited strong binding affinity to fresh washed mouse platelets but not human platelets as detected by flow cytometry using FITC-conjugated Fab (Fig. 1c, d). Immunoblotting of platelet lysate with 2B4 and 1D12 produced almost the same protein bands as those blotted with a commercialized well-documented anti-mouse GPIbα antibody (Fig. 1e). These results illustrated that 2B4 and 1D12 specifically recognize mouse GPIbα.

By using synthetic peptides, we obtained 20 purified recombinant GPIbα fragment (Additional file 3: Table S2B) to characterize the binding sites of 2B4 and 1D12. In ELISA, aa 277-290 showed the highest binding with 2B4; meanwhile, 1D12 bound to aa 41-50 (Additional file 4: Figure S2A and B), and the binding of the antibody to the peptide fragment was in a concentration-dependent manner (Fig. 1f). Interestingly, SZ2 antibody was found binding to aa 268-282 GPIbα fragment in human platelet [23]; it therefore shares the similar binding site with that of 2B4 on mouse platelets (this was confirmed in this study shown in Additional file 4: Figure S2D). In addition, AK2 antibody recognized aa 36-59 GPIbα fragment in human platelet [24], therefore also overlapping the binding site with that of 1D12 on mouse platelet. Since previous experiments showed that vWF could bind to these two binding sites in GPIbα [25], we therefore speculated that 2B4 and 1D12 could also affect vWF binding.

### Anti-mouse GPIbα monoclonal antibodies 2B4 and 1D12 inhibit vWF binding

**Fig. 2:**
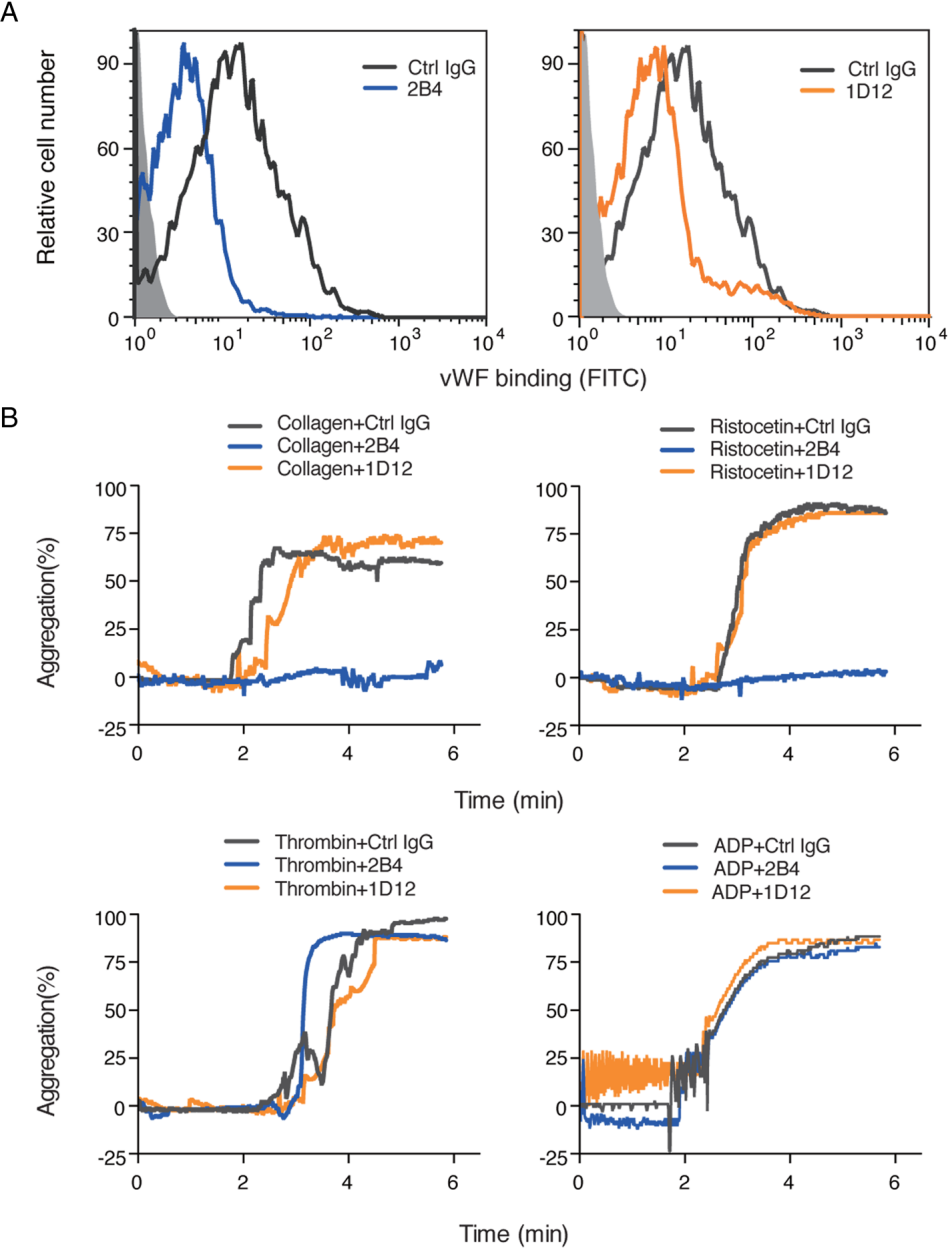
2B4 and 1D12 inhibit vWF binding. **a** The vWF binding was inhibited by 2B4 and 1D12 and detected by flow cytometry. Washed mouse platelets were incubated with 10 μg/ml 2B4 or 10 μg/ml 1D12 for 20 min, and then 2 μg/ml recombined mouse vWF was added in the presence of 1 mg/ml ristocetin. Binding of vWF was detected with FITC-conjugated mouse vWF IgG by flow cytometry and quantitated by mean fluorescence intensity. **b** 2B4 inhibited ristocetin- and collagen-induced platelet aggregation. Different agonist-induced aggregation of PRP that had been pretreated with 10 μg/ml rat IgG (negative control, gray), 10 μg/ml 2B4 (blue), and 10 μg/ml 1D12 (orange) were detected for 6 min. Agonists: ristocetin (1 mg/ml), thrombin (0.05 U/ml), ADP (10 nM), and collagen (2 μg/ml). The histograms are representative of three independent experiments

To determine whether 2B4 and 1D12 affect vWF binding, we tested by flow cytometry using recombined mouse vWF. Figure 2a showed that both 2B4 and 1D12 inhibited vWF binding when platelet was activated by ristocetin (1 mg/ml). Since ristocetin-induced platelet aggregation is associated with vWF binding [26], we next investigated platelet aggregation induced by several agonists. 2B4 significantly inhibited ristocetin-induced platelet aggregation but did not affect the aggregation induced by ADP and thrombin (Fig. 2b). In addition, collagen-induced platelet aggregation was totally inhibited by 2B4. This is supportive to a previously reported study that the collagen-vWF-GPIbα axis was critical for platelet adhesion to a damaged blood vessel and the binding to collagen could be influenced when vWF binding is inhibited [27]. It is noteworthy, however, that 1D12 had no influence on neither ristocetin- nor collagen-induced aggregation. This is reminiscent of a previously reported antibody p0p/1-5 [28] that affected vWF binding without influencing the aggregation induced by ristocetin and collagen. These might be because of the different binding epitopes. Nonetheless, our data in Fig. 2a demonstrated clearly that 2B4 and 1D12 could inhibit vWF binding.

### 2B4 and 1D12 inhibit tumor metastasis in vivo and in vitro

**Fig. 3:**
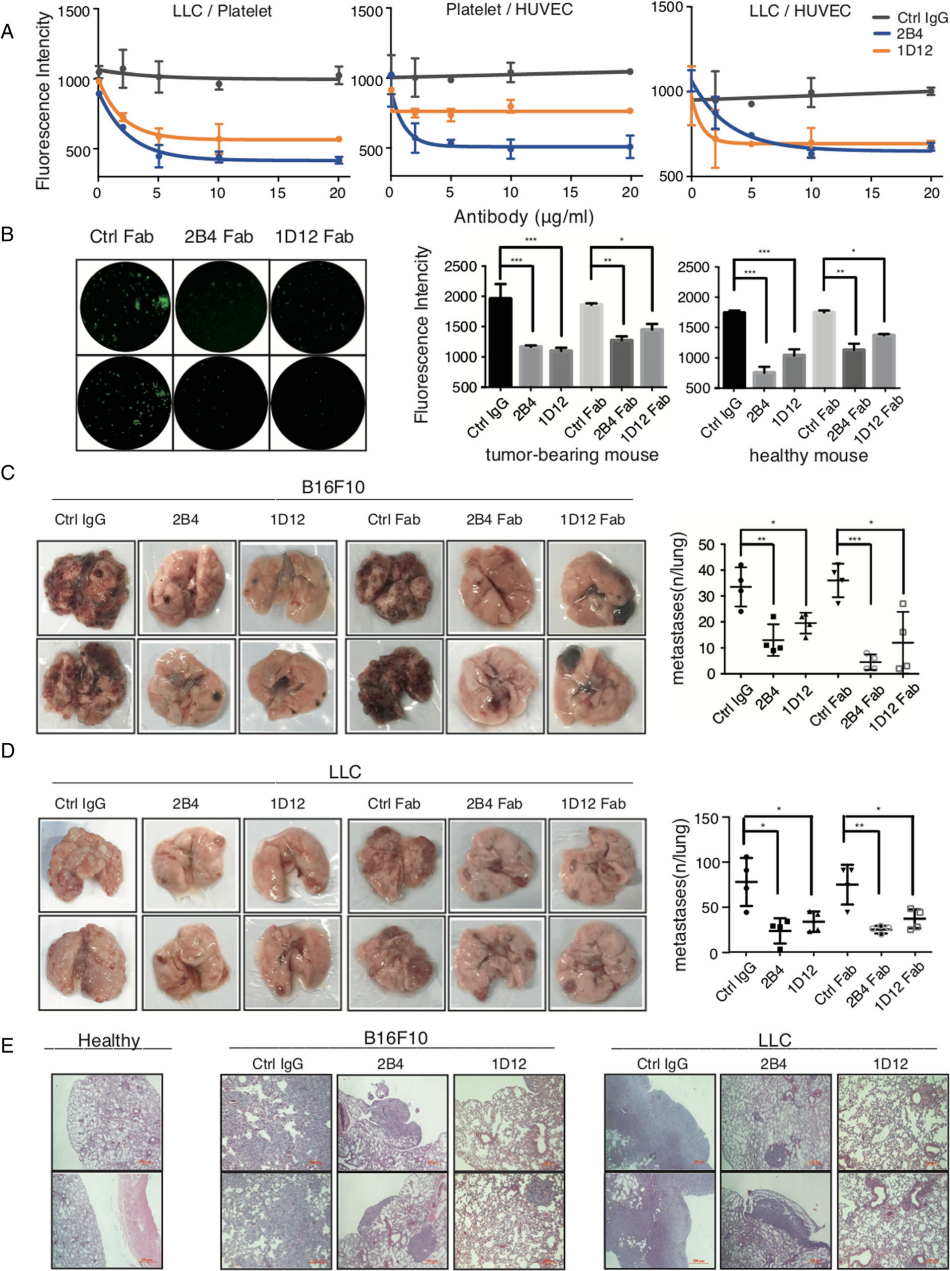
2B4 and 1D12 inhibit cancer adhesion in vitro and metastasis in vivo. **a** 2B4 and 1D12 inhibited platelet, LLC cell, and endothelial cell adhesion between each other. Effect of 2B4 (blue) and 1D12 (orange) on the adhesion of LLC with BCECF-labeled platelet (Left panel), mouse BCECF-labeled platelets with HUVECs (middle panel), and BCECF-labeled LLC cells with HUVECs (right panel) in vitro. The negative control was in the form of rat IgG (gray). **b** 2B4 and 1D12 inhibited adhesion of LLC to mouse platelets from mice with pulmonary metastasis. Washed mouse platelets were divided from tumor-bearing mouse blood and healthy mouse blood. The adhesion of LLC with BCECF-labeled platelet was detected with a fluorescence plate reader and observed under a fluorescence microscope. The negative control is in the form of rat IgG or rat Fab. Average fluorescence intensity was shown in right graphs (± SD, *P* value is indicated; *P < 0.05; **P < 0.01; ***P < 0.001). **c**, **d** Pulmonary metastasis was assessed after B16F10 and LLC injection with control IgG (50 μg/mouse), 2B4 (50 μg/mouse), 1D12 (50 μg/mouse), control Fab (50 μg/mouse), 2B4 Fab (50 μg/mouse), or 1D12 Fab (50 μg/mouse) through the lateral tail vein (*n* = 4 mice in each group). Metastasis was analyzed 14 days after injection of tumor cells. Representative examples of the lungs (one of each group) with metastatic foci were depicted. Average number of lung metastasis in each of the groups was shown in right graphs (± SD, *P* value is indicated; *P < 0.05; **P < 0.01; ***P < 0.001). **e** Representative histologic evidence from tumor sections of the different groups. Four percent of paraformaldehyde-embedded lungs of all mice were cut completely, stained with hematoxylin and eosin, and examined histologically. Representative sections of two mice from 2B4 groups, 1D12 groups, and control IgG groups were shown. Each figure is a representative of three independent experiments

To investigate whether the inhibition of vWF-GPIbα interaction was associated with tumor metastasis, we first investigated the effect of antibodies on the interaction between platelets and hypoxic-treated LCC cells in vitro. The left panel of Fig. 3a demonstrated that platelet-LLC adhesion was significantly decreased with a maximum of approximately 60% inhibition when platelets were pretreated with 2B4 or 1D12. A similar result was observed using platelets from tumor-bearing mice (Fig. 3b). Because vWF-GPIbα and vWF-collagen interactions play a critical role in the adhesion of platelets to the endothelial cells [29], we therefore reasoned that 2B4 and 1D12 could inhibit the adhesion between platelets and endothelial cells. The middle panel in Fig. 3a showed that platelet adhesion to hypoxic-treated HUVECs was reduced in a dose-dependent manner after incubation with 1D12 or 2B4. Meanwhile, the adhesion of BCECF-labeled hypoxic-treated LLC cells to hypoxic-treated HUVECs was also markedly decreased when platelets were pretreated with 2B4 or 1D12 (Fig. 3a, right panel). As expected, the negative control, normal rat IgG had no effect.

**Fig. 4:**
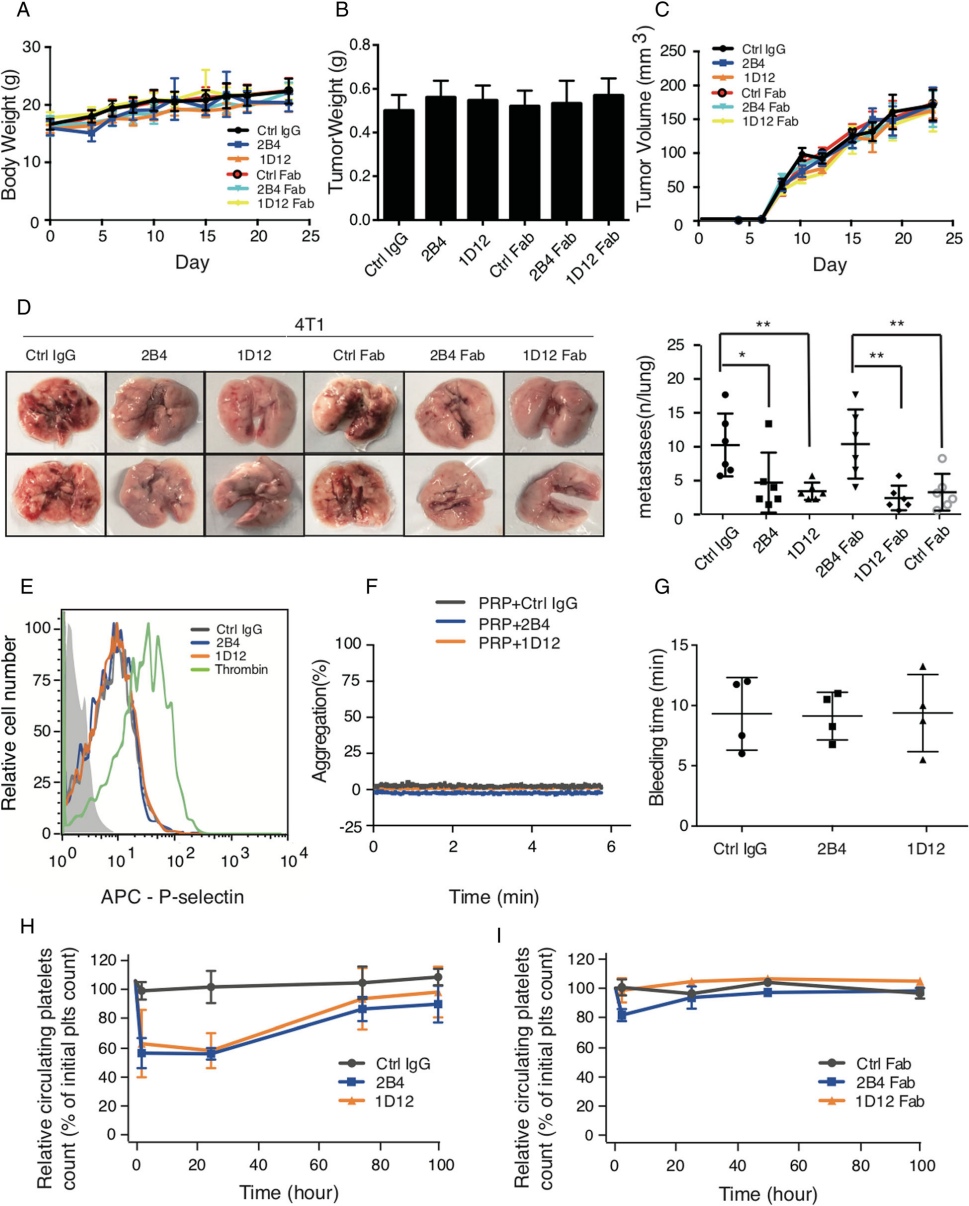
2B4 and 1D12 inhibit spontaneous metastasis but have no effect on platelet activation and hemostatic function. Effect of 2B4 (50 μg/mouse), 1D12 (50 μg/mouse), and their Fabs (50 μg/mouse) on (**a**) mice weight, (**b**) tumor weight, (**c**) tumor volume, and (**d**) pulmonary metastasis on spontaneous metastasis of 4T1 (*n* = 6 mice in each group). Metastasis was analyzed 3 weeks after injection of tumor cells. Representative examples of the lungs (one of each group) with metastatic foci were depicted. Average number of lung metastasis in each of the groups was shown in right graphs (± SD, *P* value is indicated; *P < 0.05; **P < 0.01; ***P < 0.001). **e** 2B4 and 1D12 did not affect platelet activation. Increased expression of APC-conjugated P-selectin indicated the degree of platelet activation. Washed platelets were treated with 10 μg/ml purified 2B4 (blue), 1D12 (orange), negative control (rat IgG, gray), or 0.05 U/ml thrombin (green) as positive control, then probed with APC-conjugated anti-P-selectin Ab. The florescence intensity was detected by flow cytometry. **f** 2B4 and 1D12 did not induce platelet aggregation. The aggregation of PRP pretreated with 10 μg/ml 2B4 (blue) and 10 μg/ml 1D12 (orange) was detected. Negative control was in the form of rat IgG. **g** Bleeding time did not prolonged 2 h after the injection of intact 2B4 or 1D12 (50 μg/mouse) (*n* = 4 mice in each group). **h** Platelet survival curves for mice injected with 2B4 (50 μg/mouse), 1D12 (50 μg/mouse), or negative control (normal rat IgG, 50 μg/mouse) (*n* = 6 mice in each group). **i** Platelet survival curves for mice injected with 2B4 Fab (50 μg/mouse), 1D12 Fab (50 μg/mouse), or negative control (normal rat Fab, 50 μg/mouse) (*n* = 6 mice in each group). Blood was drawn from mice at the time of antibody injection (0 h), 2 h after injection, and every 24 h following until 96 h after antibody injection. Platelet count determined by flow cytometry. The histograms are representative of three independent experiments

We further investigated the inhibitory effect of these antibodies in vivo using an experimental metastasis model with female C57BL/6J mice. As noted in Fig. 3c, the melanoma cells B16F10 co-incubated with either 2B4 or 1D12 (or their Fabs) resulted in a markedly decreased number of surface pulmonary nodules compared to the control group using ctrl IgG or its Fab. The same phenomenon was observed when using LLC cells (Fig. 3d). This finding was again confirmed by counting the metastatic lesions under microscopy using the H-E slides of the lung tissue (Fig. 3e). In addition, in vivo experimental metastasis assay with male mice also showed inhibition of tumor metastasis by antibodies (data not shown). Moreover, in the spontaneous metastasis model, the body weight and the volume of primary tumor was not be influenced by antibody injection (Fig. 4a–c), but the lung metastasis was obviously suppressed (Fig. 4d). All together, these data suggested 2B4 and 1D12 could potently inhibit the adhesion of cancer cells in vitro and metastasis in vivo.

### 2B4 and 1D12 have no effect on platelet activation and hemostatic function

Since platelet activation and subsequent clearing induced by antibodies targeting GPIbα limited the clinical application of previous platelet antibodies in suppressing cancer metastasis [18, 30], it is therefore important to investigate whether 2B4 and 1D12 could affect platelet activation and/or induce thrombocytopenia in vivo. Treatment of washed mouse platelets with 2B4 or 1D12 did not induce increased expression of P-selectin (Fig. 4e). Furthermore, the addition of 2B4 or 1D12 to PRP did not induce platelet aggregation (Fig. 4f). Injection of 2B4 or 1D12 at the dose of 50 μg/mouse did not affect tail-bleeding time (Fig. 4g). While the injection of 2B4 or 1D12 decreased almost 40% of the platelet count, that of their respective Fab fragments did not change the platelet count (Fig. 4h, i). Therefore, 2B4 and 1D12 can potentially suppress cancer metastasis without significantly affecting the number and function of platelets.

### Generation and characterization of mouse anti-human platelet GPIbα monoclonal antibody YQ3

**Fig. 5:**
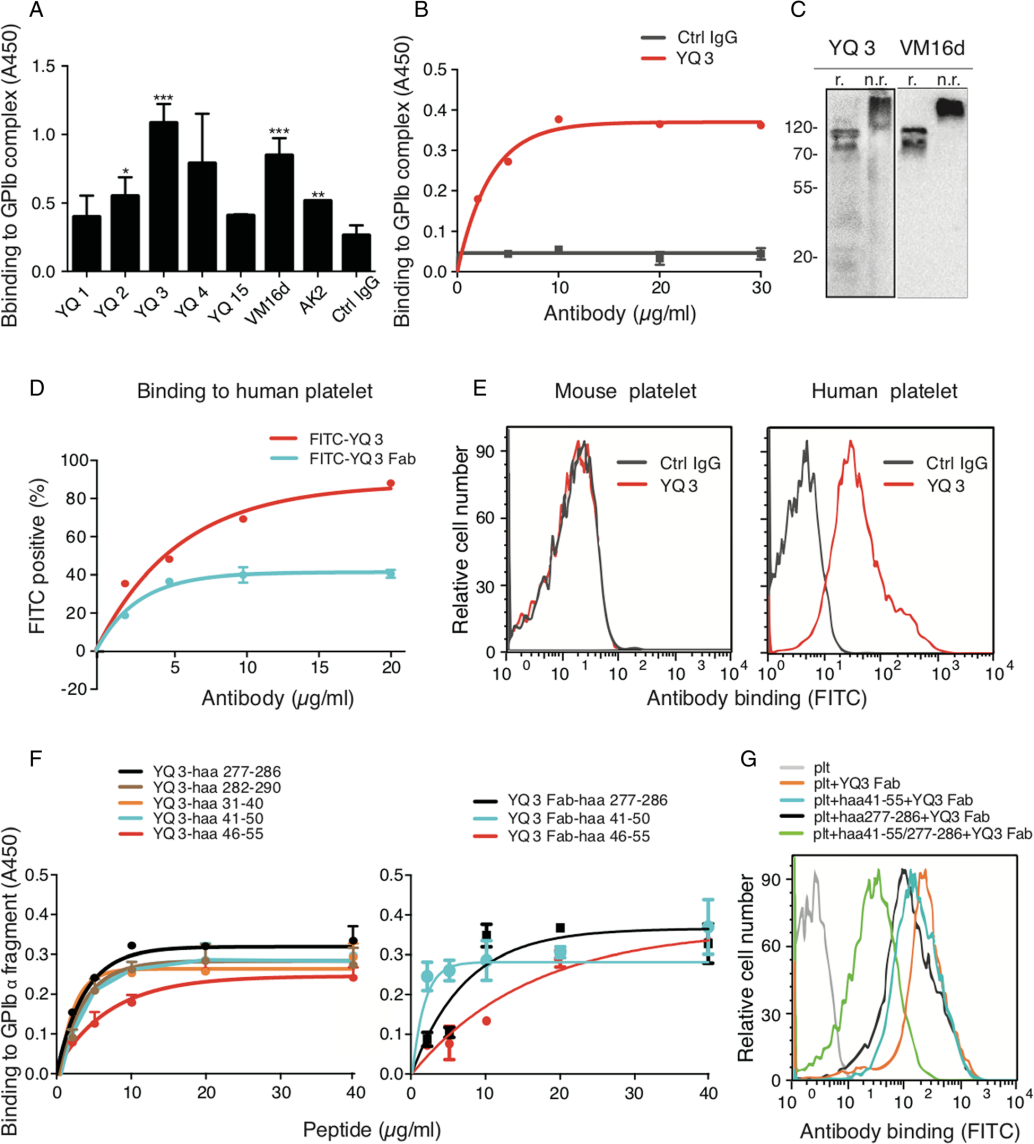
YQ3 binds specifically to human glycoprotein (GP)Ibα. **a** Binding of mouse anti-human antibodies to GPIb-IX complex was detected in ELISA. GPIb-IX was captured by anti-GPIX antibody which complex was immobilized in microtiter plates. Supernatant of hybridoma cells, each identified by the clone name, and the negative control, in the form of RPMI-1640 fetal bovine culture medium with 5 μg/ml mouse IgG, were added to the coated wells. The bound Ab was detected with HRP-conjugated rabbit anti-mouse IgG. **b** Binding of YQ3 to GPIb-IX complex was detected in ELISA. Negative control was in the form of normal mouse IgG. The bound Ab was detected with HRP-conjugated rabbit anti-mouse IgG. **c** YQ3 recognized specifically GPIbα in Western blot. **d** Binding of FITC-conjugated YQ3 or YQ3 Fab to washed human platelets was detected by flow cytometry. Washed human platelets were incubated with YQ3 and YQ3 Fab at indicated concentration. **e** YQ3 specifically bound to human platelet. Washed human and mouse platelets were incubated with 10 μg/ml FITC-conjugated YQ3. Binding of Ab was detected by flow cytometry and quantitated by mean fluorescence intensity. **f** Binding of YQ3 and its Fab to GPIbα peptide fragment was detected in ELISA. Indicated concentration of GPIbα peptide fragment was immobilized in microtiter plates. Ten micrograms per milliliter of YQ3 or YQ3 Fab was added to the coated wells. The bound Ab or Fab was detected with HRP-conjugated rabbit anti-mouse IgG. **g** The competitive binding of peptides and platelet to antibodies was detected by flow cytometry. Washed human platelets were pretreated with aa 41-55, aa 277-286, or both aa41-55 and aa277-286 before adding FITC-conjugated YQ3 Fab. The fluorescence intensity was detected in flow cytometry. Each figure or histogram is a representative of three independent experiments

Based on the above findings, we then decided to use the same approach to generate a series of mouse anti-human platelet GPIbα monoclonal antibodies to explore the role of human GPIbα in cancer metastasis (Additional file 1: Table S1B). After screening, we obtained a potent antibody, YQ3, as the best candidate for the subsequent studies. Our screening results showed that compared to other candidates, YQ3 exhibited the strongest binding affinity to GPIb-IX complex (Fig. 5a) and inhibitory effect on adhesion (Additional file 2: Figure S1D).

Further experiments illustrated that purified YQ3 (Additional file 2: Figure S1E) exhibited strong binding to immobilized GPIb-IX complex in a dose-dependent manner (Fig. 5b, *K*_d_ of YQ3 is 2.26 ± 0.37 μg/mL). Meanwhile, it specifically recognized human but not mouse platelet GPIbα (Fig. 5c–e). By using 20 purified human GPIbα fragments (Additional file 3: Table S2A) in ELISA, we found that YQ3 bound to aa 31-56 and aa 277-290 of human GPIbα in a dose-dependent manner. (Fig. 5f left panel, Additional file 4: Figure S2C). These two binding sites overlapped those of the above-mentioned two rat anti-mouse antibodies. In addition, the Fab of YQ3 was found to bind to aa 41-56 and aa 277-286 (Fig. 5f right panel, Additional file 4: Figure S2E). To further verify the accuracy of the binding sites, we conducted a competitive binding assay. Briefly, washed human platelets were pretreated with aa41-55 or aa277-286, or both peptides before adding FITC-conjugated YQ3 Fab. As expected, peptides aa 41-56 and aa 277-286 inhibited 50% binding of YQ3 Fab to platelets. When aa 41-56 or aa 277-286 was used alone, such binding was also impaired, but to a lesser degree (Fig. 5g).

### YQ3 inhibits adhesion and cancer cell-induced platelet activation

**Fig. 6:**
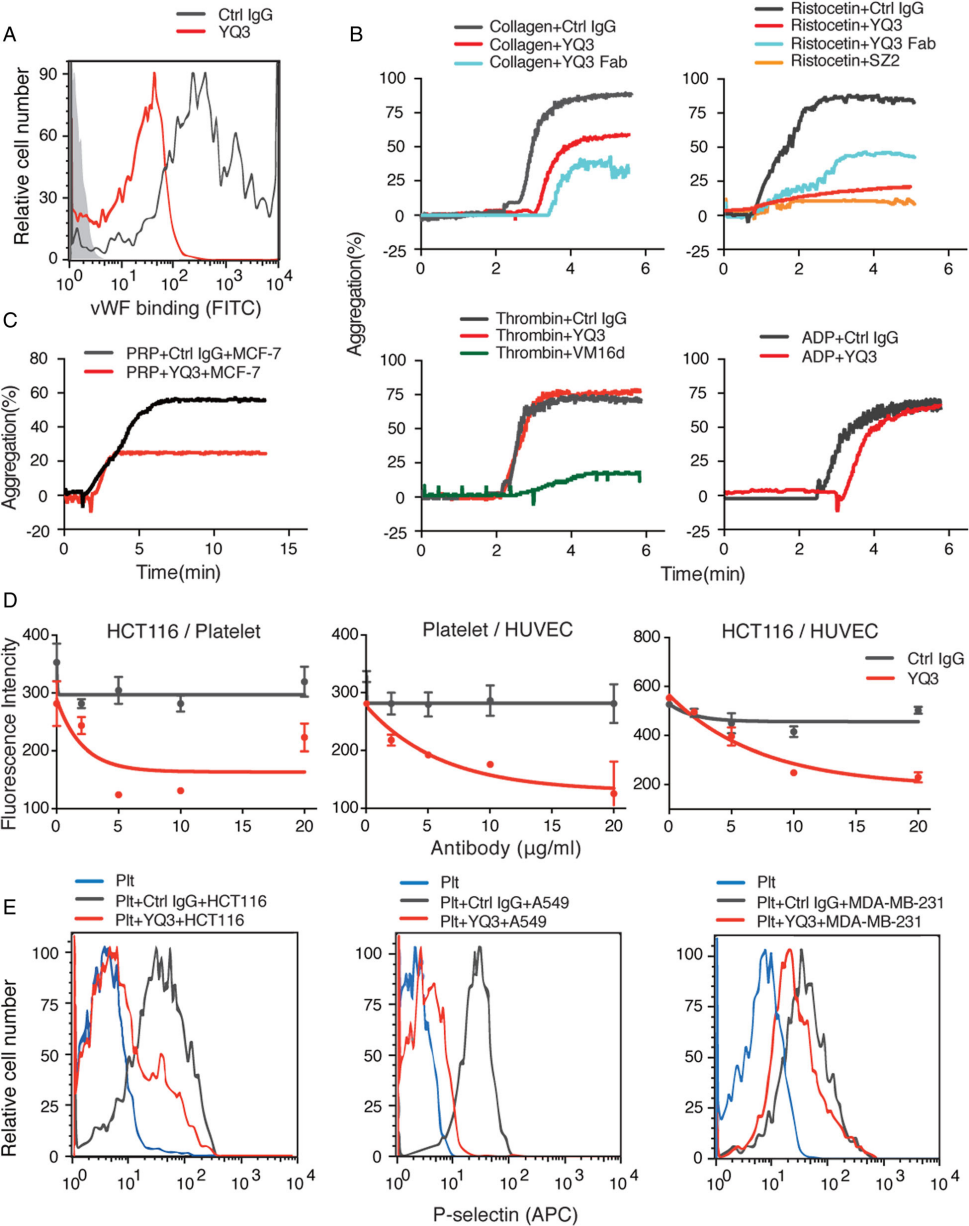
YQ3 inhibits adhesion and cancer cell-induced platelet activation. **a** The vWF binding was inhibited by YQ3 detected by flow cytometry. Washed human platelets were incubated with 2 μg/ml recombined human vWF in the presence of 1 mg/ml ristocetin. Binding of vWF was detected with FITC-conjugated human vWF IgG by flow cytometry and quantitated by mean fluorescence intensity. **b** YQ3 inhibited ristocetin- and collagen-induced platelet aggregation. Different agonist-induced aggregation of PRP that had been pretreated with 10 μg/ml mouse IgG (negative control), 10 μg/ml YQ3, or 10 μg/ml YQ3 Fab was detected for 6 min. **c** YQ3 inhibited MCF-7-induced platelet aggregation. Fifty microliters of MCF-7 cell suspension with 1 × 10^5^ cells/ml was added to PRP. Platelet had previously been incubated with 10 μg/ml YQ3 for 5 min. Aggregation was detected for 13 min. **d** YQ3 inhibited platelet, HCT 116 cell, and endothelial cell adhesion between each other. Left panel, the quantitative analysis of adhesion of HCT116 with BCECF-labeled platelet in the presence of various concentration of YQ3. Middle panel, the effect of YQ3 on the adhesion of BCECF-labeled platelets to HUVECs. Right panel, the effect of YQ3 on platelet-mediated BCECF-labeled HCT116 cells adhesion to HUVECs in vitro. **e** YQ3 inhibited tumor cell-induced platelet activation. Fifty microliters of different tumor cell (HCT116, A549, or MDA-MB-231) suspension with 1 × 10^5^ cells/ml was added to washed platelet. Platelet was pretreated with 10 μg/ml YQ3 or negative control (normal mouse IgG). Platelet activation was detected by APC-conjugated P-selectin Ab. The histograms are representative of three independent experiments

We next investigated whether YQ3 could affect vWF binding. Figure 6a showed that vWF binding induced by ristocetin in vitro was decreased when incubated with YQ3. Meanwhile, ristocetin- and collagen-induced aggregation were also significantly inhibited by YQ3 and its Fab fragment. However, ADP and thrombin-induced aggregation was not influenced (Fig. 6b).

To investigate inhibitory potential of YQ3 in cancer metastasis, we first tested YQ3 on TCIPA. Figure 6c showed that platelet aggregation induced by breast cancer cells MCF-7 was partially inhibited by the addition of YQ3. We then tested the effect of YQ3 on adhesions between platelet, tumor cells (e.g., colorectal cancer HCT116), and HUVECs. Figure 6d showed that YQ3 inhibited cell adhesion between HCT116 cells and platelets, platelets and HUVECs, and HUVECs and HCT116 cells. Similar results were observed when we used other tumor cell lines such as breast cancer MDA-MB-231 and lung cancer A549 cells (data not shown).

We then tested the effect of YQ3 on tumor cell-induced platelet activation. Different types of tumor cells (e.g., HCT116, A549, and MDA-MB-231) were incubated with platelet with or without YQ3. The expression of P-selectin was used to indicate the degree of platelet activation. Again, YQ3 was found able to inhibit tumor cell-induced platelet activation (Fig. 6e). These results together suggested that YQ3 might have the potential to inhibit metastasis.

### YQ3 inhibits the adhesion between patients’ platelets and tumor cells without accelerating platelet clearance

**Fig. 7:**
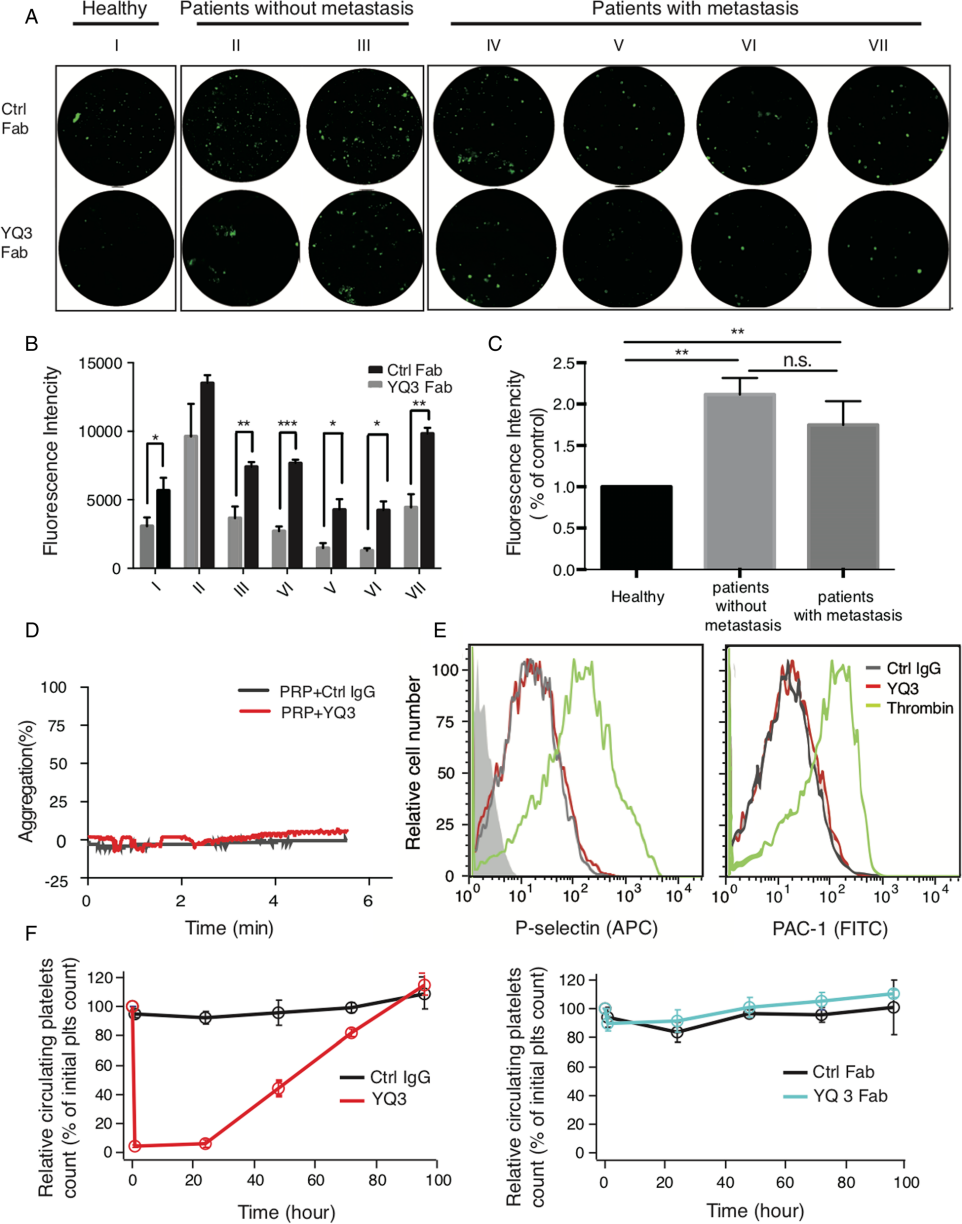
YQ3 inhibits the adhesion between patients’ platelets and tumor cells without accelerating platelet clearance. **a**, **b** YQ3 inhibited adhesion of A549 lung cancer cells to patients’ platelets. **a** The adhesion of A549 to patients’ platelets pretreated with 10 μg/ml YQ3 Fab as observed under fluorescence microscope. I: healthy person; II/III: patients without metastasis; IV/V/VI/VII: patients with metastasis. The quantitative analysis of adhesion was shown in **b**, *P* value is indicated; *P < 0.05; **P < 0.01; ***P < 0.001. **c** Expression of GPIbα on platelets that were from healthy controls, patients without metastasis, or patients with metastasis. Fluorescence intensity was detected by flow cytometry. **P < 0.01, n.s., no signifance. **d** YQ3 did not induce platelet aggregation. The aggregation of PRP pretreated with 10 μg/ml YQ3 was detected. Negative control was in the form of normal mouse IgG. **e** YQ3 did not affect platelet activation. Increased expression of P-selectin and PAC-1 indicated the degree of platelet activation. Washed human platelets were treated with 10 μg/ml purified YQ3 (red) and negative control (mouse IgG, gray) or 0.05 U/ml thrombin (green) as positive control, then probed with APC-conjugated P-selectin Ab and FITC-conjugated APC-1 Ab. The fluorescence intensity was detected by flow cytometry. **f** Platelet survival curves for hTg mice injected with YQ3 (15 μg/mouse), YQ3 Fab (15 μg/mouse), or negative control (mouse IgG or Fab, 15 μg/mouse) (*n* = 6 mice in each group). Blood was drawn from mice at the time of antibody injection (0 h) and every 24 h following until 96 h after antibody injection. Platelet count determined by flow cytometry. Each figure or histogram is a representative of three independent experiments

We next tested the effect of YQ3 using patients’ blood. Pre-incubated platelets collected from lung cancer patients (patients II and III had no metastasis; patients IV–VII presented with metastasis) with YQ3 Fab dramatically attenuated their adhesion to A549 cells (Fig. 7a, b). It is therefore supportive to our previous finding that YQ3 inhibited the adhesion between platelets and tumor cells. Moreover, while the expression levels of GPIbα were higher in lung cancer patients than in healthy controls, no difference was found between patients with metastasis and without, suggesting the expression level of GPIbα could not be correlated to the progression of cancer metastasis (Fig. 7c).

We then tested whether in patients’ blood, YQ3 could affect platelet activation or platelet count. Figure 7d demonstrated that YQ3 did not induce PRP aggregation. Moreover, treatment of washed human platelets with YQ3 did not induce activation of integrin αII_b_β_3_ or increase the expression of P-selectin (Fig. 7e). To explore the effect of YQ3 on platelet count, hTg mice were treated with YQ3 or YQ3 Fab, and platelet count was monitored over a 4-day period after injection. Figure 7f showed that the injection of YQ3 induced severe decrease of platelet count, but its Fab did not. Therefore, mouse anti-human GPIbα monoclonal antibody YQ3 will likely not have concerning bleeding complications, and it is worth a further exploration in humanized models.

## Discussion

Multiple studies have shown that the platelet GPIbα is an important receptor in the process of tumor metastasis [9, 18, 31, 32]. However, the biggest obstacle to use GPIbα inhibition for cancer treatment is potential severe bleeding complications. This is even more concerning when conventional chemotherapy is used since it almost universally affects platelet count. However, several antibodies specifically targeting platelet GPIbα and inhibiting vWF binding to platelet were reported not to influence the platelet count dramatically [33–35]. Nancy et al. investigated the anti-thrombotic effect of human GPIbα mAb 6B4 Fab fragment in vivo and found that through inhibition of the binding of vWF to GPIbα, fewer platelets were activated, resulting in decreased risk of bleeding [33]. Similarly, the anti-human vWF monoclonal antibody SZ-123 was found to inhibit vWF-collagen and vWF-platelet interactions in vivo and did not significantly prolong bleeding time [34, 35]. These findings are important considering inhibition of collagen-vWF-GPIbα axis is therefore considered as a new strategy in anti-thrombotic therapy [29]. Nevertheless, contradictory findings were reported that vWF deficiency could promote tumor metastasis instead [36]. It remains debatable whether this was due to enhanced platelet GPIbα availability that promoted metastasis in the absence of vWF. If this is the case, then inhibiting the interactions among collagen, vWF, and GPIbα might be valuable. Indeed, in this study, we have confirmed such strategy is useful with minimal effect on platelet number and function.

Compared with traditional anti-platelet drugs, which prevent thrombosis by inhibiting normal platelet function, the monoclonal antibodies developed in this study utilize a different mechanism of action. In our report, we developed two novel rat anti-mouse GPIbα monoclonal antibodies, 2B4 and 1D12, and a mouse anti-human GPIbα monoclonal antibody YQ3. These antibodies exhibited inhibitory potential on cancer metastasis by blocking the vWF-GPIbα axis without affecting platelet activation and hemostatic function. Therefore, we proved that it is possible to use antibodies to inhibit metastasis without inducing thrombocytopenia by blocking the vWF-GPIbα interaction.

Several anti-GPIbα monoclonal antibodies which inhibit vWF binding to platelet have already been developed, these include p0p/B [9, 28], SZ2 [23], AK2 [24], and VM16d [37]. Luise et al. [9] investigated the effect of Fab fragment of p0p/B, a mAb directed against the vWF-binding site on mouse GPIbα, on pulmonary metastasis. An unexpected increase in experimental metastasis after blockade of GPIbα was observed. The mechanism of p0p/B promotion on metastasis was thought that the blockade of GPIbα by p0p/B led to the decrease in platelet interaction with P-selectin, which then resulted in increased availability of P-selectin for the direct interaction of cancer cells with endothelium cells. In comparison, in our study, 1D12 and 2B4 were found to inhibit metastasis likely due to their capacity of inhibiting the vWF-GPIbα interaction. Therefore, despite using GPIbα as the same target, using antibodies binding to different sites of GPIbα, hence affecting its binding partners through the resultant spatial conformations, distinct influence on cancer metastasis could be observed.

Interestingly, the binding site of our YQ3 overlaps the previously reported monoclonal antibody SZ2, which binds to vWF-binding site aa 268-282 on mouse GPIbα [23]. Leslie et al. [38] reported previously that MCF-7-induced platelet aggregation was inhibited by 46% when tumor cells were pretreated with SZ2. However, SZ2 failed to affect the extent of platelet-LS174T cell hetero-aggregation [39]. Furthermore, the human GPIbα monoclonal antibody AK2 [24], which has an overlap-binding site aa 41-55 with YQ3, was not reported on its effect on metastasis. Based on the above reports, we tested the effect of SZ2 and AK2 on platelet-tumor cell binding. SZ2 did not affect the adhesion between patients’ platelets and tumor cells (Additional file 4: Figure S2F). In addition, AK2 also had no effect on the adhesion of platelet to HCT116 cells (data not shown). As spatial conformation is critical for function, for example, normal thrombus formation was performed primarily through tethering of GPIbα to the A1 domain of immobilized vWF^14^, different antibodies may exert different effects despite their binding to similar sites (secondary structure) on GPIbα. We therefore propose that YQ3, SZ2, and AK2 may have distinct spatial conformation upon binding to GPIbα. Meanwhile, our data did suggest such possibility: YQ3 not only inhibited vWF binding (platelet aggregation induced by ristocetin) but also platelet aggregation induced by collagen. Similarly, 1D12 and 2B4 also inhibited the aggregation induced by collagen. Therefore, likely a broader effect on collagen-vWF-GPIbα interaction played the role in promoting metastasis, which is different from the mechanism of the other platelet antibodies used in anti-thrombotic therapy. Certainly, more definitive evidence is warranted.

It is interesting that injection of YQ3 full-length antibody to hTg mice induced severe thrombocytopenia similar to the traditional anti-platelet drugs, but injection of Fab fragment alone did not. Such phenomenon might be explained by a recently proposed theory that anti-GPIbα antibodies harboring bivalent structure may exert a pulling force on platelet GPIbα by crosslinking platelets under shear flow [30]. The bivalent structure of YQ3 full-length antibody could therefore exert pulling force to induce platelet clearing, while the univalent structure of YQ3 Fab will not. The YQ3 Fab could serve as a prototype for further exploration.

Currently, metastasis inhibition potential of anti-human GPIIb-IIIa agents including oral antagonist XV454 [10], abciximab, tirofiban, and eptifibatide [40] have been investigated in murine models. However, as the key receptor in the most important and common final pathway of platelet aggregation, blockade of GPIIb-IIIa will likely influence the hemostasis and coagulation. Even though the humanized anti-GPIIIa49-66 scFv Ab A11 demonstrated significant inhibition in metastasis with prolonged bleeding time that could recover in 24 h, the precipitous drop of platelet count by about 70% is concerning [12]. In addition, cancer metastasis still cannot be maximally inhibited because metastasis can be carried out by the adherence between tumor cells and GPIbα. Novel compounds such as YQ3 need to be pursued.

Various ligands of GPIbα, such as vWF, thrombin [16], and P-selectin [15, 41], are all essential for metastasis-promoting activity of platelets, resulting in a complex role of GPIbα in the process of tumor cell metastasis. This study demonstrated that targeting the interaction among collagen, vWF, and GPIbα in cancer therapy could attenuate the metastatic potential of tumor cells. We therefore reinforced the importance of GPIbα in metastasis, as well as the great potential in suppressing metastasis via novel targeting strategies.

## Conclusion

In summary, we obtained two novel rat anti-mouse GPIbα antibodies, 1D12 and 2B4, and a mouse anti-human GPIbα antibody YQ3. All of antibodies have a potential effect on inhibition of tumor metastasis by affecting the collagen, vWF, and GPIbα interaction via binding to GPIbα aa 41-50 and aa 277-290, respectively. We therefore demonstrated the role of GPIb in promoting tumor metastasis and found a new target for the inhibition of tumor metastasis.

## Additional files

Additional file 1: **Table S1.** The screening of hybridoma cell clones. (A) Rat anti-mouse platelet clones and (B) mouse anti-human platelet clones. Binding of clones to GPIb-IX complex was detected in ELISA. GPIb-IX complex was captured by anti-GPIX antibody, which was immobilized in microtiter plates. Supernatant of hybridoma cells were added to the coated wells. The bound clone was detected with HRP-conjugated rabbit anti-rat IgG or rabbit anti-mouse IgG. Effect of clones on BCECF-labeled platelets adherence to tumor cells was recorded by fluorescence plate reader. BCECF-labeled platelets pretreated with supernatant of different hybridoma cells were added into tumor cells coated plates. The adherence between platelets and tumor cells was detected by fluorescence plate reader. (XLSX 36 kb)

Additional file 2: **Figure S1.** Screening of six rat anti-mouse GPIbα antibodies and five mouse anti-human GPIbα antibodies. (A) The quantitative analysis of adhesion of LLC cells with BCECF-labeled mouse platelets in the presence of various antibodies was measured of fluorescent intensity under fluorescence plate reader. (B) Effect of antibodies on platelet activation was detected by flow cytometry. Washed platelets were treated with hybridoma supernatant and negative control (RPMI-1640 fetal bovine culture medium with rat IgG) and then probed with APC-conjugated anti-P-selectin Ab. (C) Purified of 2B4 and 1D12 and its Fab fragments were run in 10% Bis-Tris SDS gel electrophoresis under reducing (r.) and nonreducing (n.r.) conditions. Molecular weight marker (M) was shown and labeled in kDa. (D) The quantitative analysis of adhesion of HCT116 cells with BCECF-labeled human platelets in the presence of various antibodies was measured of fluorescent intensity under fluorescence plate reader. (E) Purified of YQ3 and its Fab fragment were run in 10% Bis-Tris SDS gel electrophoresis under reducing (r.) and nonreducing (n.r.) conditions. Molecular weight marker (M) was shown and labeled in kDa on the left. *P* value is indicated; *P < 0.05; **P < 0.01; ***P < 0.001. Each figure is a representative of three independent experiments. (TIFF 8219 kb)

Additional file 3: **Table S2.** Mouse (B) and human (A) GPIbα peptides fragment sequences. (XLSX 43 kb)

Additional file 4: **Figure S2.** Characterization of antibodies’ binding sites. Mouse platelet GPIbα fragments bound to (A) 2B4 and (B) 1D12. Human platelet GPIbα fragments bound to (C) YQ3, (D) SZ2 and (E) YQ3 Fab. 20 μg/ml platelet GPIbα fragment was immobilized in microtiter plates. Ten micrograms per milliliter of antibody was added to the coated wells, respectively. (F) SZ2 did not affect adhesion of A549 lung cancer cells to patients’ platelets. The adhesion of A549 to patients’ platelets pretreated with 10 μg/ml SZ2 as observed under fluorescence microscope. I*V*/V/VI/VII: patients with metastasis. N.S.: No Significant Difference. (TIFF 8219 kb)

## Abbreviations

GPIbα: Platelet glycoprotein Ibα
mAB: Monoclonal antibodies
TCIPA: Tumor cell-induced platelet aggregation
vWF: von Willebrand factor

## References

[1] Wojtukiewicz MZ, Hempel D, Sierko E, Tucker SC (2017). Antiplatelet agents for cancer treatment: a real perspective or just an echo from the past?. Cancer Metastasis Rev.

[2] Bambace NM, Holmes CE (2011). The platelet contribution to cancer progression. J Thromb Haemost.

[3] Ikeda M, Furukawa H, Imamura H, Shimizu J (2002). Poor prognosis associated with thrombocytosis in patients with gastric cancer. Ann Surg Oncol.

[4] Monreal M, Fernandezllamazares J, Piñol M, Julian JF (1998). Platelet count and survival in patients with colorectal cancer--a preliminary study. Thromb Haemost.

[5] Symbas NP, Townsend MF, Elgalley R, Keane TE (2001). Poor prognosis associated with thrombocytosis in patients with renal cell carcinoma. British Journal of Urology International.

[6] Gücer F, Moser F, Tamussino K, Reich O (1998). Thrombocytosis as a prognostic factor in endometrial carcinoma. Gynecol Oncol.

[7] Zhang W, Dang S, Hong T, Tang J (2012). A humanized single-chain antibody against beta 3 integrin inhibits pulmonary metastasis by preferentially fragmenting activated platelets in the tumor microenvironment. Blood.

[8] Poggi A, Vicenzi E, Cioce V, Wasteson A (1988). Platelet contribution to cancer cell growth and migration: the role of platelet growth factors. Pathophysiol Haemost Thromb.

[9] Erpenbeck L, Nieswandt B, Schön M, Pozgajova M (2010). Inhibition of platelet GPIbα and promotion of melanoma metastasis. J Investig Dermatol.

[10] Amirkhosravi A, Mousa SA, Amaya M, Blaydes S (2003). Inhibition of tumor cell-induced platelet aggregation and lung metastasis by the oral GpIIb/IIIa antagonist XV454. Thromb Haemost.

[11] Trikha M, Zhou Z, Timar J, Raso E (2002). Multiple roles for platelet GPIIb/IIIa and alphavbeta3 integrins in tumor growth, angiogenesis, and metastasis. Cancer Res.

[12] Nardi M, Feinmark SJ, Hu L, Li Z (2004). Complement-independent Ab-induced peroxide lysis of platelets requires 12-lipoxygenase and a platelet NADPH oxidase pathway. J Clin Invest.

[13] Jain S, Harris J, Ware J (2010). Platelets: linking hemostasis and cancer. Arterioscler Thromb Vasc Biol.

[14] Terraube V, Marx I, Denis CV (2007). Role of von Willebrand factor in tumor metastasis. Thromb Res.

[15] Chen M, Geng JG (2006). P-selectin mediates adhesion of leukocytes, platelets, and cancer cells in inflammation, thrombosis, and cancer growth and metastasis. Arch Immunol Ther Exp.

[16] Nierodzik ML, Plotkin A, Kajumo F, Karpatkin S (1991). Thrombin stimulates tumor-platelet adhesion in vitro and metastasis in vivo. J Clin Investig.

[17] Löf A, Müller JP, Breehm MA (2017). A biophysical view on von Willebrand factor activation. J Cell Physiol.

[18] Jain S, Zuka M, Liu J, Russell S (2007). Platelet glycoprotein Ib alpha supports experimental lung metastasis. Proc Natl Acad Sci U S A.

[19] Ware J, Russell S, Ruggeri ZM (2000). Generation and rescue of a murine model of platelet dysfunction: the Bernard-Soulier syndrome. Proc Natl Acad Sci U S A.

[20] Köhler G, Milstein C, Köhler G, Milstein C (1975). Continuous cultures of fused cells secreting antibody of predefined specificity. Biotechnology.

[21] Liang X, Russell SR, Estelle S, Jones LH (2013). Specific inhibition of ectodomain shedding of glycoprotein Ibα by targeting its juxtamembrane shedding cleavage site. J Thromb Haemost.

[22] Chen W, Liang X, Syed AK, Jessup P (2016). Inhibiting GPIbalpha shedding preserves post-transfusion recovery and hemostatic function of platelets after prolonged storage. Arterioscler Thromb Vasc Biol.

[23] Cauwenberghs N, Vanhoorelbeke K, Vauterin S, Westra DF (2001). Epitope mapping of inhibitory antibodies against platelet glycoprotein Ibalpha reveals interaction between the leucine-rich repeat N-terminal and C-terminal flanking domains of glycoprotein Ibalpha. Blood.

[24] Ward CM, Andrews RK, Smith AI, Berndt MC (1996). Mocarhagin, a novel cobra venom metalloproteinase, cleaves the platelet von Willebrand factor receptor glycoprotein Ibalpha. Identification of the sulfated tyrosine/anionic sequence Tyr-276-Glu-282 of glycoprotein Ibalpha as a binding site for von Willebra. Biochemistry.

[25] Ruan CG, Du XP, Xi XD, Castaldi PA (1987). A murine antiglycoprotein Ib complex monoclonal antibody, SZ 2, inhibits platelet aggregation induced by both ristocetin and collagen. Blood.

[26] Li C, Piran S, Chen P, Lang S (2011). The maternal immune response to fetal platelet GPIbα causes frequent miscarriage in mice that can be prevented by intravenous IgG and anti-FcRn therapies. The Journal of clinical investigation.

[27] Wu D, Vanhoorelbeke K, Cauwenberghs N, Meiring M (2002). Inhibition of the von Willebrand (VWF)-collagen interaction by an antihuman VWF monoclonal antibody results in abolition of in vivo arterial platelet thrombus formation in baboons. Blood.

[28] Bergmeier W, Rackebrandt K, Schröder W, Zirngibl H (2000). Structural and functional characterization of the mouse von Willebrand factor receptor GPIb-IX with novel monoclonal antibodies. Blood.

[29] Vanhoorelbeke K, Ulrichts H, Schoolmeester A, Deckmyn H (2003). Inhibition of platelet adhesion to collagen as a new target for antithrombotic drugs. Curr Drug Targets Cardiovasc Haematol Disord.

[30] Quach ME, Dragovich MA, Chen W, Syed AK (2017). Fc-independent immune thrombocytopenia via mechanomolecular signaling in platelets. Blood.

[31] Jain S, Russell S, Ware J (2009). Platelet glycoproteinVI facilitates experimental lung metastasis in syngenic mouse models. J Thromb Haemost.

[32] Karpatkin S, Pearlstein E, Ambrogio C, Coller BS (1988). Role of adhesive proteins in platelet tumor interaction in vitro and metastasis formation in vivo. J Clin Investig.

[33] Cauwenberghs N, Meiring M, Vauterin S, van Wyk V (2000). Antithrombotic effect of platelet glycoprotein Ib-blocking monoclonal antibody Fab fragments in nonhuman primates. Arterioscler Thromb Vasc Biol.

[34] Zhao YM, Jiang M, Ji SD, He Y (2013). Anti-human VWF monoclonal antibody SZ-123 prevents arterial thrombus formation by inhibiting VWF-collagen and VWF-platelet interactions in Rhesus monkeys. Biochem Pharmacol.

[35] Zhao Y, Dong N, Shen F, Xie L (2007). Two novel monoclonal antibodies to VWFA3 inhibit VWF-collagen and VWF-platelet interactions. J Thromb Haemost.

[36] Terraube V, Pendu R, Baruch D, Gebbink MF (2006). Increased metastatic potential of tumor cells in von Willebrand factor-deficient mice. J Thromb Haemost.

[37] Mazurov AV, Vinogradov DV, Vlasik TN, Repin VS (1991). Characterization of an antiglycoprotein Ib monoclonal antibody that specifically inhibits platelet-thrombin interaction. Thromb Res.

[38] Oleksowicz L, Mrowiec Z, Schwartz E, Khorshidi M (1995). Characterization of tumor-induced platelet aggregation: the role of immunorelated GPIb and ja:math expression by MCF-7 breast cancer cells. Thromb Res.

[39] McCarty OJ, Jadhav S, Burdick MM, Bell WR (2002). Fluid shear regulates the kinetics and molecular mechanisms of activation-dependent platelet binding to colon carcinoma cells. Biophys J.

[40] Leclerc JR (2002). Platelet glycoprotein IIb/IIIa antagonists: lessons learned from clinical trials and future directions. Crit Care Med.

[41] Palumbo JS, Kombrinck KW, Drew AF, Grimes TS (2000). Fibrinogen is an important determinant of the metastatic potential of circulating tumor cells. Blood.

