# Supplementary figures and images for: Novel antibodies against GPIbα inhibit pulmonary metastasis by affecting vWF-GPIbα interaction
Source: J Hematol Oncol. 2018 Sep 17;11:117. doi: 10.1186/s13045-018-0659-4 (PMC6142402; doi:10.1186/s13045-018-0659-4)

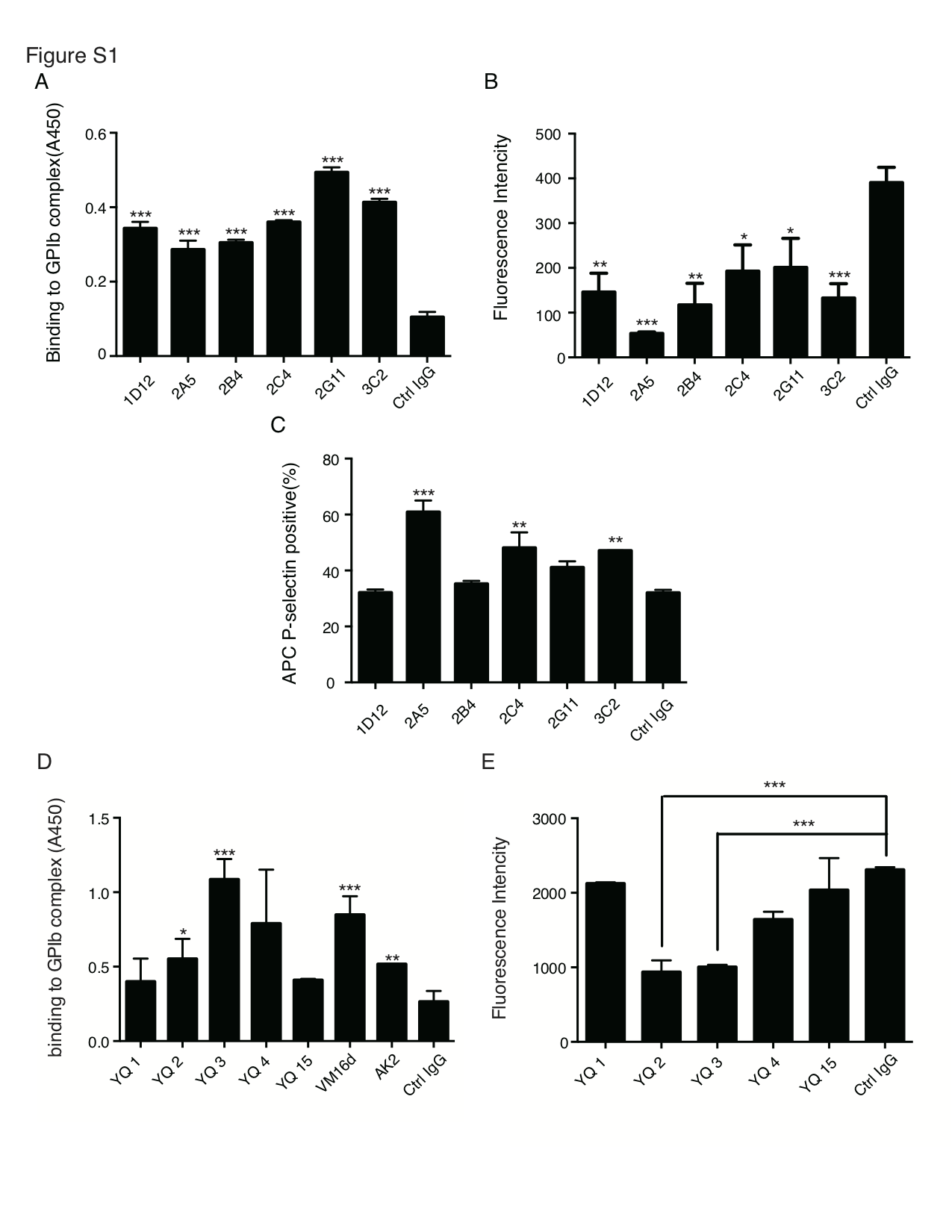

Supplement: Supplementary file 2 — Figure S1. Screening of six rat anti-mouse GPIbα antibodies and five mouse anti-human GPIbα antibodies. (A) The quantitative analysis of adhesion of LLC cells with BCECF-labeled mouse platelets in the presence of various antibodies was measured of fluorescent intensity under fluorescence plate reader. (B) Effect of antibodies on platelet activation was detected by flow cytometry. Washed platelets were treated with hybridoma supernatant and negative control (RPMI-1640 fetal bovine culture medium with rat IgG) and then probed with APC-conjugated anti-P-selectin Ab. (C) Purified of 2B4 and 1D12 and its Fab fragments were run in 10% Bis-Tris SDS gel electrophoresis under reducing (r.) and nonreducing (n.r.) conditions. Molecular weight marker (M) was shown and labeled in kDa. (D) The quantitative analysis of adhesion of HCT116 cells with BCECF-labeled human platelets in the presence of various antibodies was measured of fluorescent intensity under fluorescence plate reader. (E) Purified of YQ3 and its Fab fragment were run in 10% Bis-Tris SDS gel electrophoresis under reducing (r.) and nonreducing (n.r.) conditions. Molecular weight marker (M) was shown and labeled in kDa on the left. P value is indicated; *P < 0.05; **P < 0.01; ***P < 0.001. Each figure is a representative of three independent experiments. (TIFF 8219 kb) [file 13045_2018_659_MOESM2_ESM.tiff]

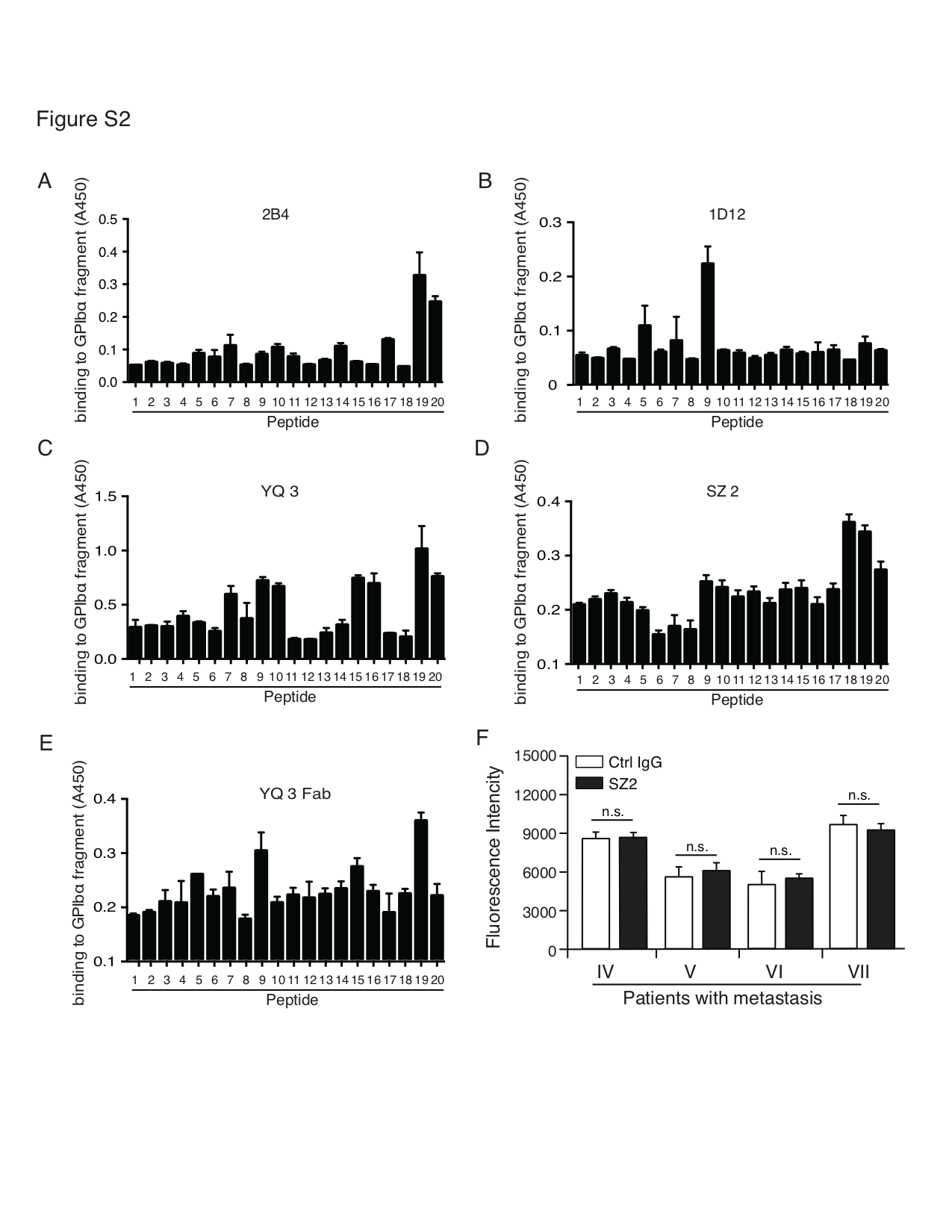

Supplement: Supplementary file 4 — Figure S2. Characterization of antibodies’ binding sites. Mouse platelet GPIbα fragments bound to (A) 2B4 and (B) 1D12. Human platelet GPIbα fragments bound to (C) YQ3, (D) SZ2 and (E) YQ3 Fab. 20 μg/ml platelet GPIbα fragment was immobilized in microtiter plates. Ten micrograms per milliliter of antibody was added to the coated wells, respectively. (F) SZ2 did not affect adhesion of A549 lung cancer cells to patients’ platelets. The adhesion of A549 to patients’ platelets pretreated with 10 μg/ml SZ2 as observed under fluorescence microscope. IV/V/VI/VII: patients with metastasis. N.S.: No Significant Difference. (TIFF 8219 kb) [file 13045_2018_659_MOESM4_ESM.tiff]
